# A Case of Basal Cell Carcinoma Arising in Congenital Triangular Alopecia

**DOI:** 10.7759/cureus.90808

**Published:** 2025-08-23

**Authors:** Allison Bai, Saman Namazian, Erika Elliott

**Affiliations:** 1 Dermatology, University of Illinois College of Medicine Peoria, Peoria, USA; 2 Dermatology, Tufts University School of Medicine, Boston, USA; 3 Dermatology, Tufts Medical Center, Boston, USA

**Keywords:** alopecia, basal cell carcinoma, congenital triangular alopecia, scalp, sonic hedgehog pathway

## Abstract

Congenital triangular alopecia (CTA) is a rare, non-scarring form of hair loss that typically presents in childhood. We present the case of a 48-year-old woman with CTA who developed basal cell carcinoma (BCC) within the right temporal alopecic patch, representing, to our knowledge, the first documented instance of malignant transformation to BCC in this condition. This case highlights the importance of evaluating long-standing alopecic areas for potential malignancy. Although the mechanisms linking CTA to carcinogenesis are not fully understood, factors such as alterations in the local immune microenvironment, increased ultraviolet exposure, and genetic predispositions may play a role in this rare transformation.

## Introduction

Congenital triangular alopecia (CTA), also referred to as temporal triangular alopecia, is a benign, rare, localized, and non-scarring form of alopecia that typically emerges in early childhood [1]. CTA appears as a triangular or oval patch of hair loss in the temporal region, where normal terminal hairs are replaced by vellus hairs, and the condition remains stable over time [2]. The underlying etiology remains unknown, though it is thought to involve localized defects in follicular development [3]. The prevalence of CTA is extremely low, with estimates of approximately 0.11% in dermatology clinic populations and fewer than 200 cases reported worldwide [4]. In contrast, basal cell carcinoma (BCC) is the most common human malignancy, with an annual incidence ranging from 1,000 to 1,500 cases per 100,000 adults in the United States and Europe, and over two million new cases diagnosed annually in the United States alone [5]. BCC typically arises in areas of chronic ultraviolet (UV) exposure and is driven by aberrant activation of the sonic hedgehog signaling pathway [6].

Despite the high incidence of BCC and its well-established development in chronically sun-exposed skin, the occurrence of BCC within the stable patches of CTA has not been previously reported. This case report, to our knowledge the first to describe BCC arising within a CTA lesion, raises important questions regarding potential pathogenic mechanisms linking these conditions and underscores the need for careful evaluation of long-standing alopecic areas for malignant transformation.

## Case presentation

**Figure 1:**
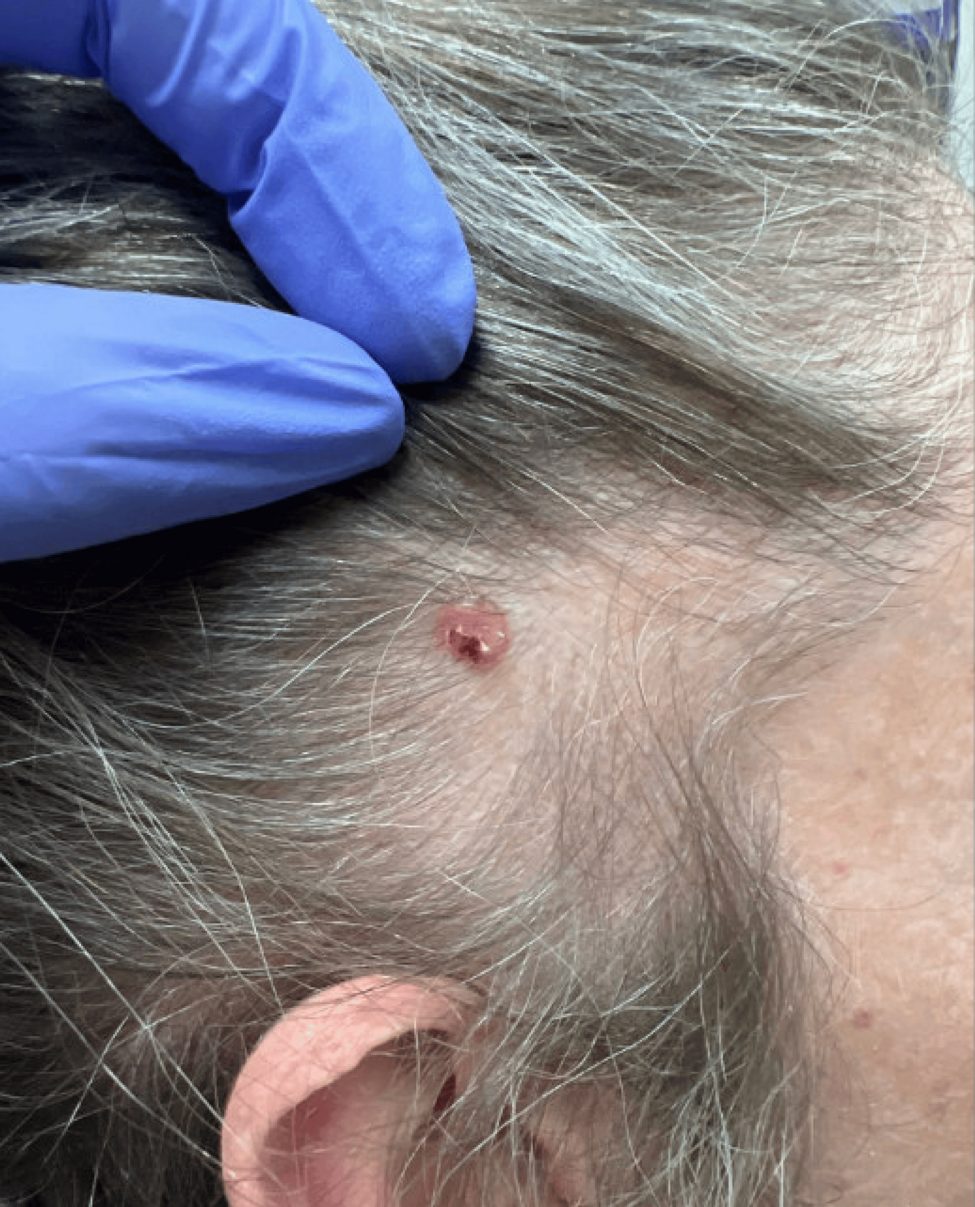
A 0.6 × 0.5 cm pink, friable papule with a blood crust located at the 7 and 8 o'clock positions within the alopecic patch on the right temple

**Figure 2:**
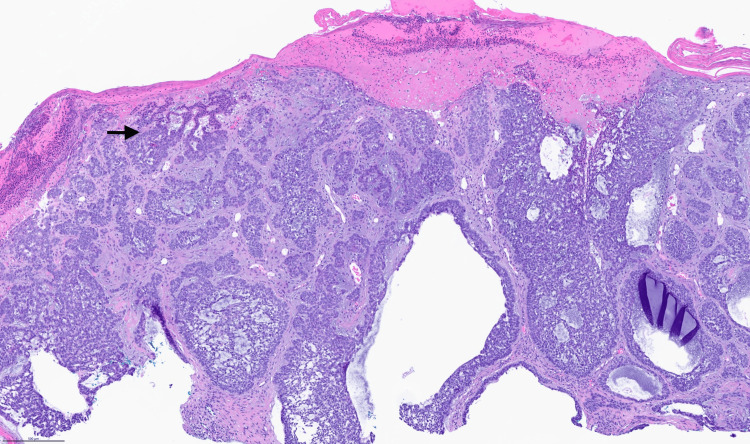
Nodular and focal infiltrative type basal cell carcinoma (black arrow) with nests of basaloid cells with peripheral palisading of nuclei and cords of basaloid cells that extends to the deep margin (H&E 2x)

A 48-year-old woman presented to Dermatology for evaluation of a lesion on her right temple that had been intermittently bleeding and fluctuating in size for the past seven months. Physical examination revealed well-defined triangular patches of hair thinning in the bilateral frontotemporal regions, along with a 0.6 × 0.5 cm pink, friable papule with a blood crust located at the 7 and 8 o'clock positions within the right alopecic patch (Figure 1). The patient reported that her hair loss had remained stable since childhood and she believed it to be due to frequently wearing a tight ponytail. Dermoscopy showed arborizing vessels within the right temple papule, raising concern for BCC, as well as normal follicular openings and vellus hairs surrounded by terminal hairs at bilateral temples. Shave biopsy of the right temple papule confirmed a diagnosis of nodular and focal infiltrative BCC with extension to the deep margin (Figure 2). The lesion was managed with Mohs micrographic surgery.

## Discussion

This case represents the first documented instance of BCC arising within a CTA patch. This rare presentation prompts consideration of potential pathogenic mechanisms and underscores the importance of clinical vigilance when monitoring longstanding alopecic areas.

Several factors may contribute to this rare occurrence. One potential contributing factor is the absence of terminal hairs in CTA, which eliminates the natural photoprotective barrier provided by pigmented hair shafts. Terminal hairs, especially pigmented ones, significantly reduce UV radiation exposure to basal keratinocytes by attenuating both UVA and UVB wavelengths [5]. Without this protection, UV transmission to the epidermis increases substantially, resulting in cumulative DNA damage such as formation of cyclobutane pyrimidine dimers and 6-4 photoproducts, which are mutagenic lesions implicated in skin carcinogenesis [6]. Chronic UV exposure is the primary environmental risk factor for BCC through direct DNA damage and mutations in tumor suppressor genes including p53 and PTCH1, as well as suppression of local immune surveillance [7]. Areas lacking hair coverage are more vulnerable to UV-induced damage, and studies have demonstrated that hairless skin develops UV-related BCC earlier and more frequently than haired skin [8-9].

Beyond the loss of photoprotection, CTA is distinguished from the normal scalp by several genetic and microenvironmental features that may contribute to tumorigenesis. Genetically, CTA is hypothesized to result from paradominant inheritance or postzygotic mosaicism leading to localized hair follicle abnormalities [10-11]. Although the exact molecular pathways remain undefined, these genetic alterations may disrupt normal follicular development and local tissue homeostasis, potentially influencing the susceptibility to malignant transformation. The altered microenvironment within alopecic patches may also influence tumor development. Hair follicles normally maintain immune privilege during their active growth phase by downregulating antigen presentation and producing immunosuppressive signals, thereby protecting themselves from immune attack. When this immune privilege collapses, as observed in conditions such as alopecia areata, a robust local inflammatory response ensues, characterized by the infiltration of cytotoxic T cells and other immune effectors [12]. However, this inflammatory milieu has not been linked directly to increased tumor susceptibility and is more commonly associated with tissue damage and scarring. Consequently, while the reduction in follicular density inherent to CTA may alter local immune dynamics and contribute to chronic low-grade inflammation, current evidence does not support a direct causal relationship between loss of immune privilege and BCC development. Instead, the pathogenesis of BCC in this setting likely involves a complex interplay of enhanced UV-induced DNA damage, altered immune responses, and possible genetic predispositions.

Regarding other instances of BCC arising in alopecic conditions, reports have documented BCC developing in scalp regions previously treated with radiotherapy for tinea capitis, where chronic alopecia and cutaneous atrophy were present [13-14]. These radiation-associated BCCs often show distinct genetic and epigenetic features compared to BCCs arising in typical sun-exposed skin, supporting the role of local microenvironmental changes in tumorigenesis. BCC has also been reported in trauma-related and keloid scars, which share characteristics of follicular loss [15-16]. In other forms of alopecia like aplasia cutis congenita (ACC), despite congenital scarring and absence of hair follicles, invasive malignancy remains exceedingly rare, with only precancerous changes documented and no confirmed cases of BCC [17]. Population-based studies of alopecia areata, however, have not demonstrated an increased risk of skin cancers [18-19]. These findings underscore the complexity and variability of tumor risk across different alopecia subtypes.

From a clinical perspective, these considerations underscore the value of early monitoring of atypical alopecic lesions. Prompt biopsy of suspicious lesions within CTA is important, as early detection and surgical management remain the mainstays of management [4]. Treatment decision is guided by location, size, histologic subtype, and recurrence. For instance, BCC located in high-risk anatomic sites like the scalp and face or on cosmetically sensitive areas, as well as those that are large, recurrent, or belong to more aggressive histologic subtypes like infiltrative, micronodular, morpheaform, or basosquamous, should be treated with Mohs surgery. BCC located in lower risk areas, however, may be managed with standard excision with margins or curettage and electrodesiccation. Nonsurgical management with topical therapies like imiquimod and 5-fluorouracil, photodynamic therapy, or radiation may be used for low-risk BCC in individuals who are poor surgical candidates, but these approaches have higher recurrence rates and less reliable margins. Relevantly, curettage and electrodesiccation should be avoided in areas with terminal hair growth, such as the scalp, as BCC may extend along follicular structures, increasing the risk of incomplete removal and recurrence [5]. Patient education regarding realistic outcomes and the importance of sun protection is essential.

## Conclusions

This rare case of BCC arising within CTA underscores the importance of heightened awareness among clinicians, as even benign-appearing, stable alopecic patches warrant periodic evaluation. Factors such as increased UV susceptibility due to absent terminal hairs, altered local immune dynamics, and potential genetic predispositions may contribute to tumorigenesis in these areas. Ongoing surveillance, patient education on sun protection, and further research into the underlying molecular processes will enhance understanding and management of this condition.

## References

[1] Trakimas C, Sperling LC, Skelton HG, Smith KJ, Buker JL (1994). Clinical and histologic findings in temporal triangular alopecia. J Am Acad Dermatol.

[2] Wu WY, Otberg N, Kang H, Zanet L, Shapiro J (2009). Successful treatment of temporal triangular alopecia by hair restoration surgery using follicular unit transplantation. Dermatol Surg.

[3] Assouly P, Happle R (2010). A hairy paradox: congenital triangular alopecia with a central hair tuft. Dermatology.

[4] Leung AK, Barankin B (2016). Incidence of congenital triangular alopecia. An Bras Dermatol.

[5] Schmults CD, Blitzblau R, Aasi SZ (2023). Basal Cell Skin Cancer, Version 2.2024, NCCN Clinical Practice Guidelines in Oncology. J Natl Compr Canc Netw.

[6] Bale AE, Yu KP (2001). The hedgehog pathway and basal cell carcinomas. Hum Mol Genet.

[7] Lacour JP (2002). Carcinogenesis of basal cell carcinomas: genetics and molecular mechanisms. Br J Dermatol.

[8] Jans J, Garinis GA, Schul W (2006). Differential role of basal keratinocytes in UV-induced immunosuppression and skin cancer. Mol Cell Biol.

[9] Pellegrini C, Maturo MG, Di Nardo L, Ciciarelli V, Gutiérrez García-Rodrigo C, Fargnoli MC (2017). Understanding the molecular genetics of basal cell carcinoma. Int J Mol Sci.

[10] Xu J, Weng Z, Arumugam A (2014). Hair follicle disruption facilitates pathogenesis to UVB-induced cutaneous inflammation and basal cell carcinoma development in Ptch(+/-) mice. Am J Pathol.

[11] Ong JS, Seviiri M, Dusingize JC (2023). Uncovering the complex relationship between balding, testosterone and skin cancers in men. Nat Commun.

[12] Happle R (2003). Congenital triangular alopecia may be categorized as a paradominant trait. Eur J Dermatol.

[13] Yamazaki M, Irisawa R, Tsuboi R (2010). Temporal triangular alopecia and a review of 52 past cases. J Dermatol.

[14] Anzai A, Wang EH, Lee EY, Aoki V, Christiano AM (2019). Pathomechanisms of immune-mediated alopecia. Int Immunol.

[15] Cardoso JC, Ribeiro IP, Caramelo F, Tellechea O, Barbosa de Melo J, Marques Carreira I (2021). Basal cell carcinomas of the scalp after radiotherapy for tinea capitis in childhood: a genetic and epigenetic study with comparison with basal cell carcinomas evolving in chronically sun-exposed areas. Exp Dermatol.

[16] Cardoso JC, Ribeiro IP, Caramelo F, Tellechea O, Barbosa de Melo J, Marques Carreira I (2021). Multiple basal cell carcinomas of the scalp after radiotherapy: genomic study in a case with latency period over 80 years. Am J Dermatopathol.

[17] Ünverdi ÖF, Yücel S (2020). Basal cell carcinomas in trauma-related scar tissue: a rare case series. Adv Skin Wound Care.

[18] Goder M, Kornhaber R, Bordoni D, Winkler E, Haik J, Tessone A (2016). Cutaneous basal cell carcinoma arising within a keloid scar: a case report. Onco Targets Ther.

[19] Itin PH, Bargetzi MC (2000). Aplasia cutis congenita with precancerous transformation - the first case. Why do these scars never develop invasive tumors?. Eur J Dermatol.

